# P01-046 – Membrane aspects of Hemostasis disorders at ATYPIC

**DOI:** 10.1186/1546-0096-11-S1-A49

**Published:** 2013-11-08

**Authors:** P Ghazaryan, A Zakharyan, N Mkrtchyan

**Affiliations:** 1 Yerevan State Medical University after M. Heratsi, Hematology Centre After Prof. R. Yeolyan, MH RA, Yerevan, Armenia

## Introduction

The increasing trend of atypical form of FMF among the Armenian population is one of the actual problems in the Armenian medicine. Hence, it induced and promoted the necessity of studying molecular mechanisms for correction of disturbed metabolic processes and for elaboration of new methods at pathogenic therapy of FMF. The specific features of the clinical symptoms in atypical forms of FMF give the rise of complications at differential diagnosis.

## Objectives

In this research the informativity of clinical-laboratory and biochemical indicators has been studied. Also the relations between individual phospholipids of biomembranes and leuko/erythroid cells during atypical FMF were investigated.

## Methods

The intensity of 14C-glycerol and 14C-glucose incorporation was studied in vitro in the contents of individual phospholipids of erythrocytes and lymphocytes membranes at children with atypical FMF. The phospholipids were fractionated by thin layer chromatography.

## Results

The substantial increment in the myeloid cell number was observed in all investigated patients. The leuko/erytroid sells relation was 4:1 instead of normal 3:1. The erythroid cell maturation index was low. The number of leukocytes was high in all patients. The basophilic erythronormoblasts predominated over polychromatic and oxyphilic ones. In all patients the expressed thrombocytosis and megakaryocytosis accompanied with active platelet formation were described. It is established that FMF is characterized by a sharp increase of ^14^C-glucose incorporation rate in the lysophosphatidylcholines (LPC) with simultaneous decrease of rate for incorporation in the contents of phosphatidylcholines (PC) and sphyngomyelines. It is observed an increase of activity of phospholipase A_2_ and the reduction of the activity of glycerolkinase and glycerol phosphate dehydrogenase. Also an increase of LPC/PC relation coefficient and decrease of PC/phosphatic acid relation were established.

## Conclusion

Apparently the revealed changes are inherent atypical FMF. The membrane aspects of hemostasis disorders mechanisms at atypical FMF are discussed.

## Disclosure of interest

None declared.

